# Continuous Monitoring of Heart Rate Variability in Free-Living Conditions Using Wearable Sensors: Exploratory Observational Study

**DOI:** 10.2196/53977

**Published:** 2024-08-07

**Authors:** Pooja Gaur, Dorota S Temple, Meghan Hegarty-Craver, Matthew D Boyce, Jonathan R Holt, Michael F Wenger, Edward A Preble, Randall P Eckhoff, Michelle S McCombs, Hope C Davis-Wilson, Howard J Walls, David E Dausch

**Affiliations:** 1 Research Triangle Institute Research Triangle Park, NC United States

**Keywords:** heart rate variability, physiological monitoring, wearable sensors, smartwatch, PPG, photoplethysmography, monitoring, physiological, heart rate, wearable, wearables, sensor, sensors, observation study, wearable devices, devices, remote monitoring, community, data platform, data collection, health risk

## Abstract

**Background:**

Wearable physiological monitoring devices are promising tools for remote monitoring and early detection of potential health changes of interest. The widespread adoption of such an approach across communities and over long periods of time will require an automated data platform for collecting, processing, and analyzing relevant health information.

**Objective:**

In this study, we explore prospective monitoring of individual health through an automated data collection, metrics extraction, and health anomaly analysis pipeline in free-living conditions over a continuous monitoring period of several months with a focus on viral respiratory infections, such as influenza or COVID-19.

**Methods:**

A total of 59 participants provided smartwatch data and health symptom and illness reports daily over an 8-month window. Physiological and activity data from photoplethysmography sensors, including high-resolution interbeat interval (IBI) and step counts, were uploaded directly from Garmin Fenix 6 smartwatches and processed automatically in the cloud using a stand-alone, open-source analytical engine. Health risk scores were computed based on a deviation in heart rate and heart rate variability metrics from each individual’s activity-matched baseline values, and scores exceeding a predefined threshold were checked for corresponding symptoms or illness reports. Conversely, reports of viral respiratory illnesses in health survey responses were also checked for corresponding changes in health risk scores to qualitatively assess the risk score as an indicator of acute respiratory health anomalies.

**Results:**

The median average percentage of sensor data provided per day indicating smartwatch wear compliance was 70%, and survey responses indicating health reporting compliance was 46%. A total of 29 elevated health risk scores were detected, of which 12 (41%) had concurrent survey data and indicated a health symptom or illness. A total of 21 influenza or COVID-19 illnesses were reported by study participants; 9 (43%) of these reports had concurrent smartwatch data, of which 6 (67%) had an increase in health risk score.

**Conclusions:**

We demonstrate a protocol for data collection, extraction of heart rate and heart rate variability metrics, and prospective analysis that is compatible with near real-time health assessment using wearable sensors for continuous monitoring. The modular platform for data collection and analysis allows for a choice of different wearable sensors and algorithms. Here, we demonstrate its implementation in the collection of high-fidelity IBI data from Garmin Fenix 6 smartwatches worn by individuals in free-living conditions, and the prospective, near real-time analysis of the data, culminating in the calculation of health risk scores. To our knowledge, this study demonstrates for the first time the feasibility of measuring high-resolution heart IBI and step count using smartwatches in near real time for respiratory illness detection over a long-term monitoring period in free-living conditions.

## Introduction

### Background

The use of wearable sensors for monitoring health status has grown in popularity, in applications ranging from fitness to illness management [1]. Wearable sensors have the potential to provide continuous, near–real-time monitoring of human physiology with minimal discomfort during daily life activities [2]. Among wearable sensors, commercial-off-the-shelf devices, such as smartwatches and smart rings, that integrate heart rate (HR), blood oxygen, and activity monitors are popular devices for use in real-world physiological monitoring because of their already broad owner base and form factor that is compatible with long-term wear [3].

Heart rate variability (HRV) provides insight into the interplay between sympathetic and parasympathetic branches of the autonomic nervous system in response to external stressors [4]. HRV measurement using photoplethysmography (PPG) sensors, such as those embedded in smartwatches, has been tested against and found to correspond with gold-standard electrocardiogram (ECG) measurements [5]. Although PPG- and ECG-derived HRV metrics have generally good agreement, PPG-based measures can differ from ECG-based measures in the presence of physical activity, cold exposure, and other factors [6-8].

Wearable sensors provide an opportunity for personalized detection of health changes relative to an individual’s baseline state, which can differ from normalized or population-level reference values [9-11]. Interbeat interval (IBI) time-series data can be used to derive HR and HRV metrics. Changes in a person’s HR and HRV over time can signal illness, infection, and other health conditions [12-14]. Analysis of HR-based measures recorded by smartwatches and smart ring sensors has shown that these measures can indicate the presence of respiratory infections, such as influenza and COVID-19, before symptoms start [15-19].

One COVID-19 detection study retrospectively analyzed data from smartwatch users over a period of several months using HR and step summary data provided by the device vendor [18]. Another retrospective study used summary HR, HRV, respiration rate, temperature, and activity data recorded from smart ring users, although HR, HRV, and respiration rate were only available during sleep periods [19]. A prospective COVID-19 monitoring study of health care workers focusing primarily on existing smartwatch users collected vendor-provided HRV metrics over a period of 5 months [17]. Although vendor-provided HRV measures can be useful to monitor health trends, high-resolution IBI and activity data are desirable to enable transparent, open-source calculation of a full set of HRV metrics and their use in a broad range of algorithms. A long-term COVID-19 monitoring study of active military personnel retrieved average HR and step-counts data from the device-vendor cloud platform for offline processing, but the availability of these data through the cloud was typically delayed by 24-48 hours relative to the time of device recording [15].

### Goals of the Study

Unlike the aforementioned studies, we report results from participants in the community under free-living conditions who were recruited independently of their smartwatch ownership or use status. That is, we include results from participants who may or may not have been familiar with smartwatch and accompanying mobile phone app use prior to involvement in this study. The validity of continuous monitoring using 4 wearable sensors in a simulated free-living environment compared with 2 reference devices found that HR accuracy was generally high when not confounded by physical activity [2]. In this study, we used 1 wearable device to collect data in actual free-living environments over a monitoring period of 8 months.

Additionally, the data presented in this paper included each IBI recorded by PPG-based sensors, along with step counts, blood oxygen level, and respiration rate. The high-resolution data were processed prospectively in real time using open-source calculations to yield HR and HRV metrics. Study-provided Garmin Fenix 6 smartwatches paired to iOS and Android smartphones running a custom mobile app were used for long-term continuous monitoring. We explored the application of these tools to the monitoring of a cohort of 59 individuals and reported observations related to the study protocol and data set.

The objectives for the study were to (1) quantify the quality of data (data missingness and artifacts) collected in free-living conditions, (2) present examples of HR and HRV-derived metrics during a baseline healthy period, (3) use the healthy baseline as the foundation for the standardization of HRV metrics and qualitatively assess if the multivariable anomaly score (health risk score) shows promise as an indicator of viral respiratory illness, and (4) demonstrate the data collection, metrics extraction, and analysis through an automated processing pipeline for prospective monitoring. The health risk score is predicated on a difference between physiological metrics relative to the healthy baseline period, where we implement activity matching to compare current and baseline metrics within similar activity bins. Activity matching is an uncommon feature of this type of data analysis, as most device vendors do not provide metrics corrected for the level of activity.

Of particular interest in the study was the response to airborne pathogens, such as COVID-19, influenza, and other respiratory viruses, self-reported by participants through health survey questionnaires. Health risk scores exceeding a predefined threshold were checked for corresponding symptom or illness reports. Conversely, reports of viral respiratory illnesses in health survey responses were checked for corresponding changes in health risk scores to qualitatively assess the score as an indicator of acute respiratory health anomaly.

We demonstrated the use of an open-source, modular platform for the collection of high-resolution, raw data directly from smartwatch devices that bypass vendor analytics and cloud services in real-world conditions. In testing this platform, we followed participants’ experiences with using this system and gathered feedback to improve functionality and usability. The data collection and analysis system can be applied to large-scale studies and can be paired with other choices for devices and algorithms.

## Methods

### Study Design

To investigate the real-world use of HRV monitoring through an automated data collection and processing pipeline in the context of prospective illness detection, we conducted an exploratory observational study combining smartwatch data and health symptoms reported by each individual through a mobile phone app. Participants wore smartwatch devices during normal daily activity and completed surveys of health symptoms. This study reports results from data collected over a period of 8 months.

### Ethical Considerations

The human participant research protocol was reviewed and approved by the RTI International Institutional Review Board (STUDY00022001) and the US Army Medical Research and Development Command Office of Human Research Oversight (E02867.5a). Participants were recruited as a convenience sample from local law enforcement and public health departments. The consent process emphasized that participation was voluntary and that participants could withdraw at any time without penalty. Informed consent was provided by all participants. Personal identifying information was stored in a secured file and kept separate from data collected once enrolled in the study. All smartwatch and survey data were deidentified and labeled by a participant identifier for analysis. The smartwatch was offered to participants as study compensation contingent upon their compliance in providing smartwatch and survey data at least 60% of the time (excluding any technical issues).

### Wearable and Smartphone Devices

Participants were issued Garmin Fenix 6 smartwatches and were offered the use of a Samsung Galaxy A12 study phone or the option to use their phone to pair the watch with the smartphone app for data collection. The SIGMA+ (S+) Health mobile app compatible with iOS and Android devices was used for syncing data from the smartwatch and uploading files to the study database. Data were transferred from the watch to the phone using Bluetooth and from the phone to the cloud-based database using Wi-Fi or cellular connectivity. The data collection architecture is described by Temple et al [20].

### Protocol

Study demographic and health symptom questionnaires were collected and managed using REDCap (Research Electronic Data Capture; Vanderbilt University) [21,22]. At the time of study enrollment, participants self-reported demographic information including age, sex, height, weight, race, Hispanic or Latino status, influenza vaccination status, COVID-19 vaccination and booster status, and the presence of any of the following underlying medical conditions: chronic lung disease, moderate to severe asthma, serious heart condition, immunocompromised, severe obesity (BMI of ≥40 kg/m^2^), cancer, diabetes, chronic kidney disease undergoing dialysis, liver disease, or smoker.

Participants completed a daily health symptom survey accessible via a link from the S+ Health app to a REDCap questionnaire. The questionnaire consisted of symptom severity ratings and a text field for additional notes (eg, stressful events and illness details). Symptoms were rated on a scale of 0 (no symptoms) to 3 (severe symptoms) for the following categories: allergies, runny nose, sore throat, cough, shortness of breath, fever, fatigue, headache, body ache, loss of taste and smell, and gastrointestinal symptoms. A symptom score was calculated from a sum of the 11 symptom values reported in the survey.

Participants were asked to wear smartwatches continuously during normal daily activities to the extent possible. Participants were requested to sync data between the smartwatch and the S+ Health app at least twice per day. A study coordinator was available to assist participants with technical support during the study and to note any feedback shared by participants on using the smartwatch and the S+ Health app. At the close of the study, participants were invited to complete a survey on their experience.

### Data Collection and Processing

#### Data Collection

Health symptom survey response data were downloaded from REDCap and processed to extract a total symptom score (ie, the sum of the 11 individual symptoms that were rated on a scale of 0 to 3, with 3 indicating severe symptoms) and the self-reported presence of influenza, COVID-19, or other illness. Diagnostic testing and reporting were not strictly required, and laboratory test results were not always available to verify illness reports. Any other self-reported health events provided as survey comments were also noted.

IBI, step count, respiration rate, and blood oxygenation data recorded by the Garmin watches were synced to the S+ Health app and uploaded to the AWS cloud (Amazon Web Services, Inc) for storage and computational processing. The analysis in this study focused on step count and IBI measurements. IBI data included (ideally) the interval between each individual heartbeat. Step counts were reported every 1 minute.

#### Data Quality

To quantify data volume and data artifacts, raw data fraction (RDF), valid data fraction (VDF), and artifact data fraction (ADF) were defined. Data were averaged into 5-minute epochs based on time of collection within a 24-hour period, with a maximum of 288 epochs per day (24 hours per day×12 epochs per hour). The RDF represents the ratio of the number of unique data point timestamps received to the total number of data points possible during the monitoring period, in this case, the number of measured epochs relative to the maximum of 12 epochs per hour (hourly measure) or 288 epochs per day (daily measure).

Subsets of these measured epochs were labeled as valid if at least 2 step counts were measured and at least 60 valid IBI data points (>0.3 and <1.5 seconds) were measured. Two step count readings were required to ensure that the watch was powered on for at least 60 seconds (ie, the S+ app acquires a one-step count reading every minute). A total of 60 valid IBI points were required to ensure good data quality for calculating HRV during resting states (ie, at least 1 minute of data in each 5-minute epoch is needed for capturing accurate low-frequency responses). A total of 60 valid IBI points in each epoch were required to calculate a representative HR during higher levels of activity when motion artifacts can be significant. The VDF was computed as the ratio of the number of valid epochs to the maximum of 12 epochs per hour (hourly measure) or 288 epochs per day (daily measure). ADF was computed as the ratio of the number of data points removed during data cleaning to the total number of data points in the monitoring period for each data type. A binary daily survey data fraction (SDF) was computed to quantify survey reporting compliance.

#### Data Processing

Data were processed to compute “base metrics” and “standardized metrics.” Base metrics included step counts averaged in 5-minute and 30-minute windows, and HR and HRV metrics calculated from 5-minute windows of the IBI time series, including HR, log of high-frequency power (HF), log of low-frequency power (LF), LF/HF, median IBI, SD of IBI, and root mean square of successive IBI differences [23].

The HR and HRV metrics were then standardized using a *z* score calculation relative to “matched” historic healthy data. Metric standardization was performed to minimize the impact of differences from individual to individual and to incorporate activity matching to minimize the impact of physical activity level in the comparison of metrics. Data were “matched” using the 5- and 30-minute step counts by binning into resting (0 step per minute), highly active (140+ steps per minute), and all other step counts within ±30% (or at least 10 steps per minute). Each data point was compared to past data points (“baseline”) at a similar (“matched”) activity level and a measure of the deviation—*z* score—was calculated for IBI, HF, and LF metrics. We required that the current data be well separated from the baseline data (eg, by at least 24 hours) that it was matched with because a person’s current health state is strongly correlated with their recent (eg, within the past hour) health state. We further required that the baseline exclude data points that were previously marked as anomalous (eg, elevated risk score). We assumed that people were healthy for the first week of data collection unless otherwise noted.

**Figure 1:**
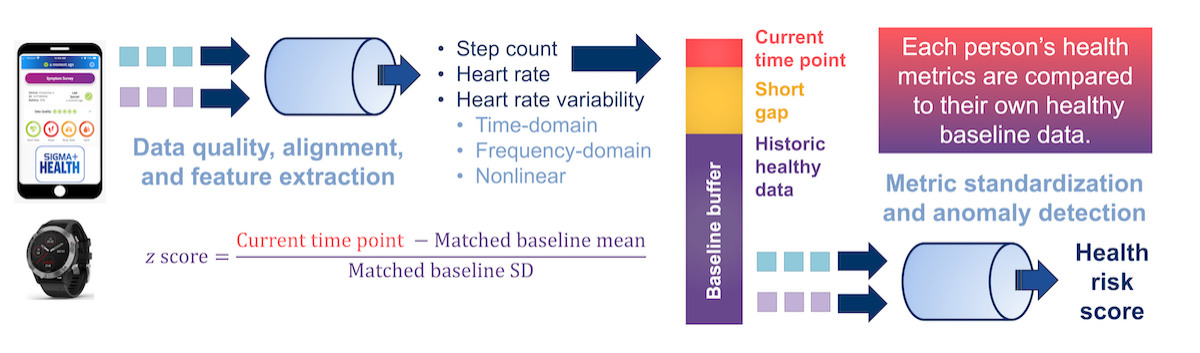
Data collection and algorithm workflow. The smartwatch is paired to a smartphone app, and data are uploaded to the data server. Data cleaning corrects sensor-specific artifacts and removes periods where data quality is poor or there is not enough data to make a representative calculation. Metrics are standardized by computing a z score, which corrects for physical activity using an individual’s healthy baseline data and eliminates between-person variation. The output of the algorithm is a risk score.

We smoothed both the standardized baseline and current data to remove rapid changes from short-term stressors using a 1-hour moving average. We calculated the Mahalanobis distance [24] between the current and healthy baseline smoothed, standardized data to obtain the health risk score. The risk score was initially developed in an influenza-challenge study in which participants were monitored using wearable ECG and physical activity sensors and tested for infection status [16]. Figure 1 illustrates the data processing workflow.

Data metrics and risk scores were stored in a Postgres database. Further details of the operation of the S+ Health app, data flow from watches to SyncHubs to data servers, and the data processing methodology are described by Temple et al [20]. Algorithms, data processing, and visualization were implemented in Python using open-source packages [25-31].

## Results

### Cohort Demographics

Health symptoms, illness, and watch data collected from January through August 2023 are reported for 59 participants. The cohort consisted of 17 female participants and 42 male participants with characteristics shown in Table 1. A total of 5 participants left the study early, and in the last 15 weeks of data collection, an additional 7 participants joined.

**Table 1:** Participant characteristics of a convenience sample of 59 individuals who provided smartwatch and health survey data between January 2023 and the end of August 2023.

Characteristics			Male (n=42)		Female (n=17)
Age (years), median (IQR)			41 (32-50)		42 (36-54)
BMI (kg/m^2^), median (IQR)			30 (27-35)		31 (28-31)
Underlying medical condition, n (%)			6 (14)		1 (6)
**Vaccination, n (%)**					
	Influenza	16 (38)		10 (59)	
	COVID-19	31 (74)		17 (100)	
	COVID-19 booster	18 (43)		14 (82)	
**Race, n (%)**					
	Asian	1 (2)		0 (0)	
	Black or African American	0 (0)		1 (6)	
	White	40 (95)		15 (88)	
	Two or more	0 (0)		1 (6)	
	Other	1 (2)		0 (0)	
Hispanic or Latino		4 (10)		2 (12)	
**Occupation, n (%)**					
	Law enforcement	29 (69)		5 (29)	
	Public health	11 (26)		7 (41)	
	Other	2 (5)		5 (29)	
**Smartphone operating system, n (%)**					
	iOS	23 (55)		10 (59)	
	Android	19 (45)		7 (41)	

### Data Collection and Processing

**Figure 2:**
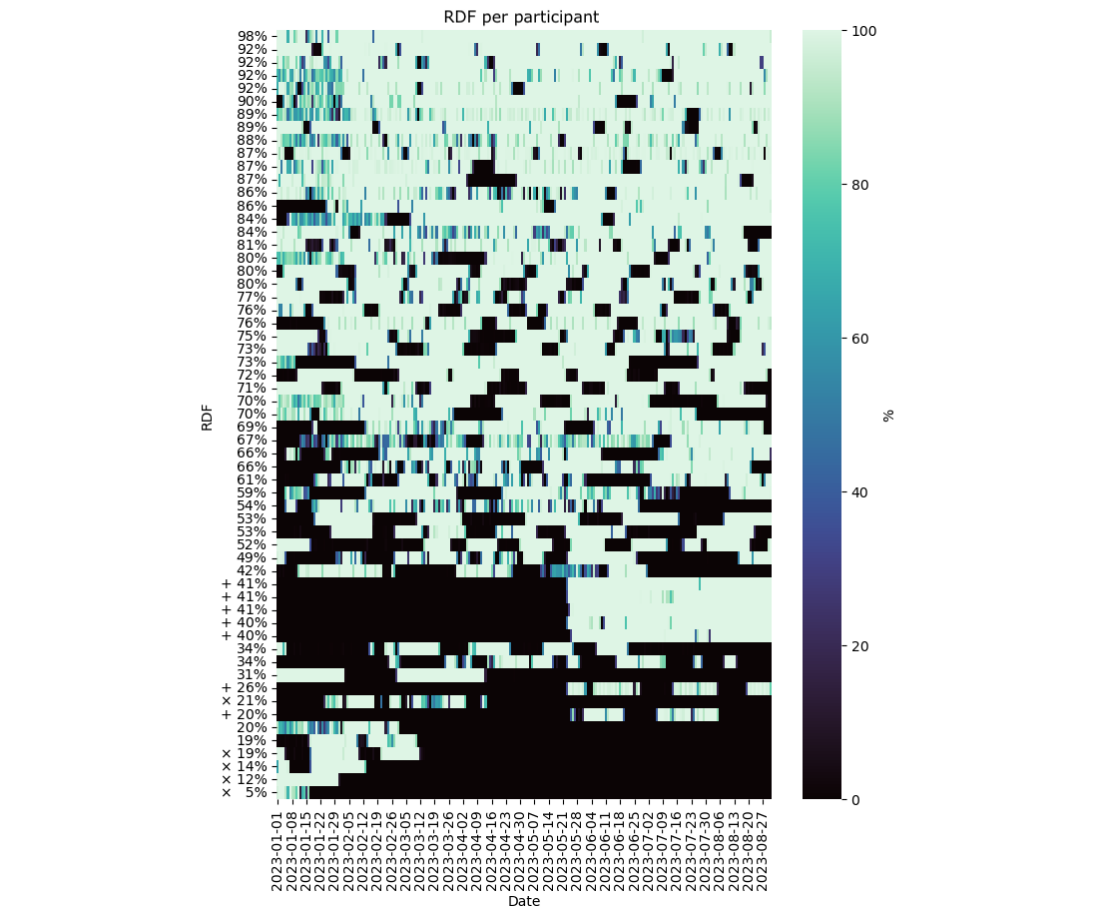
RDF per participant for a convenience sample of 59 individuals who provided smartwatch data between January 2023 and the end of August 2023. Labels on the y-axis indicate the average RDF over the 8-month monitoring period per participant. Each row illustrates daily RDF values per participant. Symbols × indicate participants who left the study and + indicate participants who joined in the last 15 weeks of the study. RDF: raw data fraction.

Figure 2 shows the raw data collected for the 8-month monitoring period, which includes periods of no data from participants who either left the study or joined partway through. To present a more complete picture of the data, we report characteristics both over the whole 8-month period and over the last 15 weeks, excluding the 5 participants who left the study.

**Figure 3:**
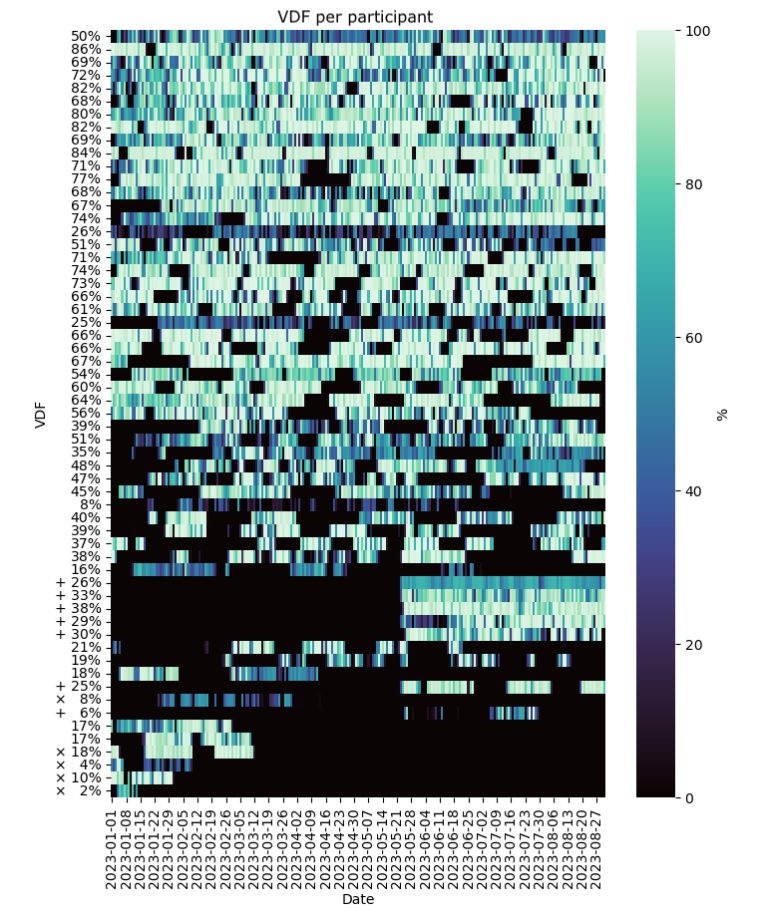
VDF per participant for a convenience sample of 59 individuals who provided smartwatch data between January 2023 and the end of August 2023. Labels on the y-axis indicate the average VDF over the 8-month monitoring period per participant. Each row illustrates daily VDF values per participant. The participant order along the y-axis corresponds to the y-axis participant ordering shown in Figure 2. Symbols × indicate participants who left the study and + indicate participants who joined in the last 15 weeks of the study. VDF: valid data fraction.

**Figure 4:**
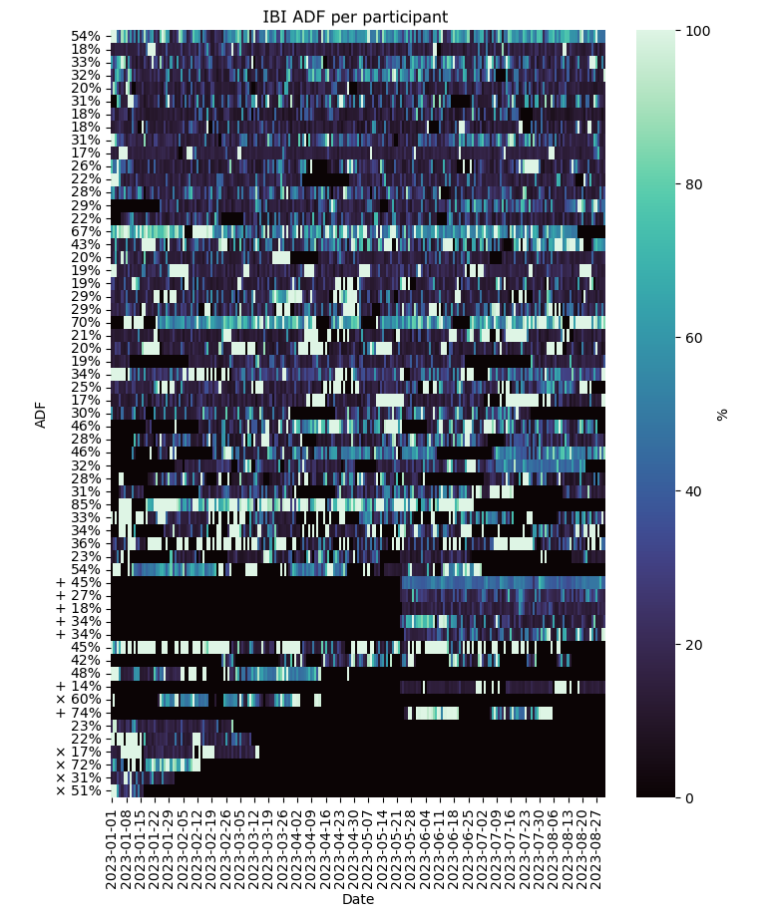
IBI ADF per participant for a convenience sample of 59 individuals who provided smartwatch data between January 2023 and the end of August 2023. Labels on the y-axis indicate the average ADF calculated from the data measured over the 8-month monitoring period per participant. Each row illustrates daily ADF values per participant. The participant order along the y-axis corresponds to the y-axis participant ordering shown in Figure 2. Symbols × indicate participants who left the study and + indicate participants who joined in the last 15 weeks of the study. ADF: artifact data fraction; IBI: interbeat interval.

The median value across participants of the average RDF over the monitoring window and the IQR are shown in Table 2. The subset of raw data that met validity criteria is shown in Figure 3, with median and IQR values reported in Table 2. From the raw data measured, we quantified the IBI ADF per participant (Figure 4). Because the ADF was calculated relative to the measured data and no value was computed during data gaps, we only report values over the 8-month period in Table 2.

**Figure 5:**
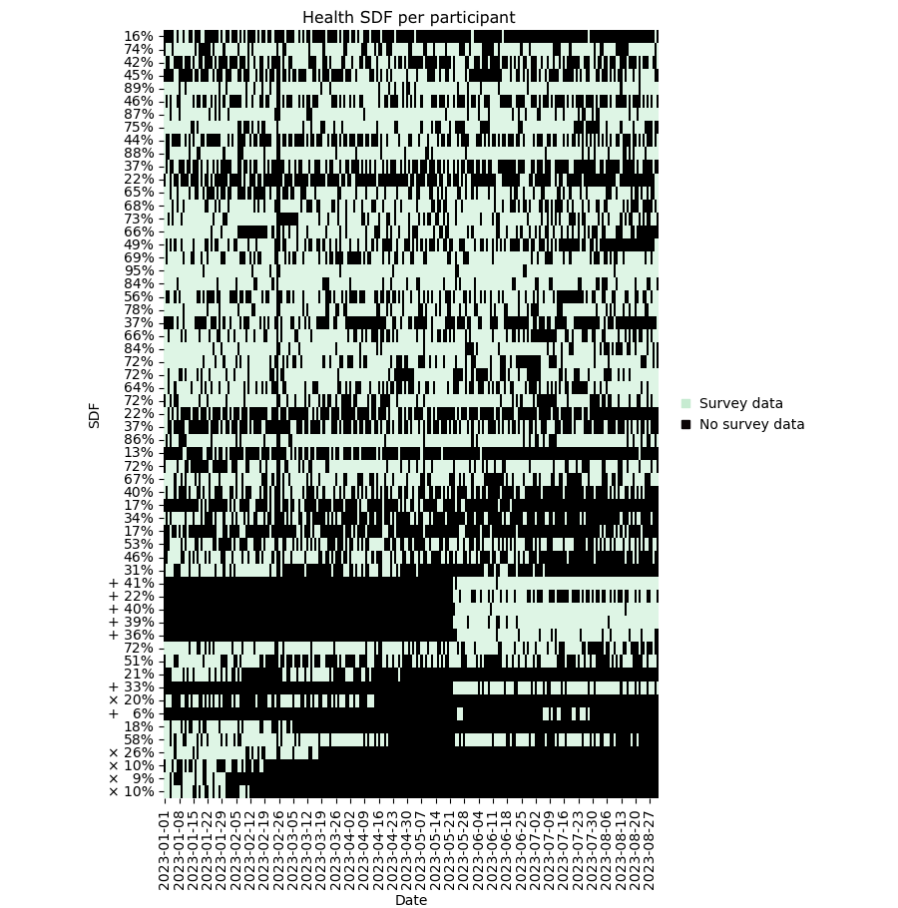
Self-reported health symptoms and illness SDF per participant for a convenience sample of 59 individuals who provided data between January 2023 and the end of August 2023. Labels on the y-axis indicate the percentage of daily survey reports submitted relative to the number of days in the 8-month monitoring period per participant. The color map indicates symptom survey reports submitted per day per participant. The participant order along the y-axis corresponds to the y-axis participant ordering shown in Figure 2. Symbols × indicate participants who left the study and + indicate participants who joined in the last 15 weeks of the study. SDF: survey data fraction.

Health symptom SDF is shown in Figure 5 and described in Table 2. Participant data are plotted in the same order along the y-axis in Figures 2-5 to facilitate comparisons between RDF, VDF, ADF, and SDF per participant. The average RDF and submitted survey percentages are not necessarily similar among participants (ie, a relatively high RDF may or may not correspond to a relatively high survey response rate).

**Table 2:** Raw, valid, IBI^a^ artifact, and survey data fraction characteristics measured from data collected from 59 individuals between January 2023 and the end of August 2023. Metrics are reported over the whole 8-month period and over a 15-week period at the end of the study corresponding to when the last 7 participants were enrolled.

Metric	Eight-month period (%), median (IQR)	Last 15 weeks (%), median (IQR)
RDF^b^	70 (41-84)	81 (60-91)
VDF^c^	48 (26-68)	60 (40-73)
ADF^d^	30 (21-42)	—^e^
SDF^f^	46 (28-72)	58 (26-75)

^a^IBI: interbeat interval.

^b^RDF: raw data fraction.

^c^VDF: valid data fraction.

^d^ADF: artifact data fraction (calculated for IBI metric).

^e^Not available.

^f^SDF: survey data fraction.

### Data Recording Challenges

During the 8-month monitoring period, 6 participants reported smartwatch-related issues and were offered replacement devices. Some participants experienced difficulty using the mobile phone app to upload data from the smartwatch. A total of 10 mobile phone app updates (combined iOS and Android) were released to mitigate data-syncing issues, including updates to the Garmin software development toolkit, and to improve user experience. Individual help was offered to participants who required assistance in updating the app on their phones. Additional challenges to data recording included a dislike of wearing the smartwatch, reported by 1 participant, and a prolonged loss of a charging cable, reported by another participant. Technical issues with either the smartwatch or app and diminished study engagement negatively impacted the data and survey responses shown in Figures 2 and 5.

### Participant Feedback

A participant experience survey was distributed at the end of the monitoring period. Of the 43 (73%) participants who completed the survey, 16 (37%) identified as being not at all familiar with using a smartwatch prior to joining this study. Regarding the smartwatch, 30 (70%) responded that the watch was a good size, 7 (16%) responded that it was not easy to use, and the most liked features of the watch were access to health data (n=15, 35%), activity tracking (n=9, 21%), and sleep tracking (n=8, 19%). Issues with data syncing and mobile app functionality were the most commonly reported problems.

**Figure 6:**
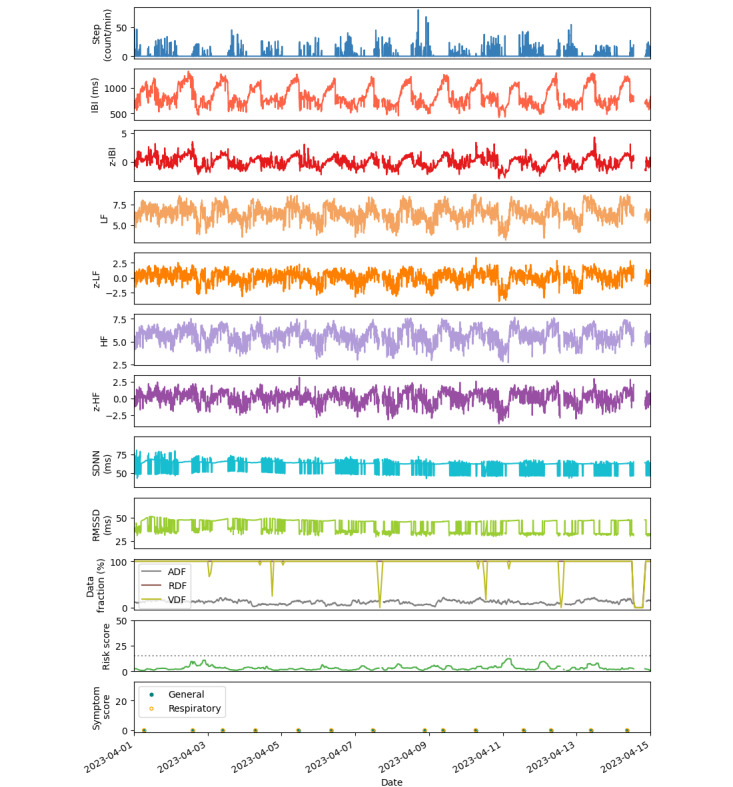
Example metrics measured from a participant over a period of 2 weeks in April 2023. All values were calculated in 5-minute epochs except symptom scores, which were calculated from self-report of individual symptoms. RDF and VDF are averaged by hour. The risk score remained below the threshold (dotted gray line) and symptom scores were zero for general and respiratory symptom categories throughout the 2 weeks. The risk scores were detected from analysis of heart rate variability-derived and activity metrics. The symptom scores were calculated from symptom ratings on a scale of 0 (no symptoms) to 3 (severe symptoms) for the following categories: allergies, runny nose, sore throat, cough, shortness of breath, fever, fatigue, headache, body ache, loss of taste and smell, and gastrointestinal symptoms. ADF: artifact data fraction (calculated for IBI metric); HF: log of high-frequency power; IBI: interbeat interval; LF: log of low-frequency power; RDF: raw data fraction; RMSSD: root mean square of consecutive heartbeat intervals; SDNN: standard deviation of IBI; Step: step count; VDF: valid data fraction; z: standardized metric.

Figure 6 shows example base and standardized HRV metrics, IBI ADF, and risk scores calculated in 5-minute epochs and hourly averages of RDF and VDF extracted from 2 weeks of a cleaned data set. Health survey responses during this time window did not report illness or symptoms. The metrics are shown as a representation of healthy baseline data (ie, *z* scores above –3 and no reported symptoms). These data also exemplify high smartwatch wear compliance and symptom survey compliance, with occasional short gaps in continuous monitoring.

### Cohort Observations

We explored the correspondence of reported symptoms or illness with the occurrence of elevated risk scores based on a threshold value of 15. This is the threshold value for which the algorithm demonstrated high sensitivity and no false alarms in the previous influenza-challenge study [16]. During the 8-month monitoring period of this study, 29 elevated score events were noted, of which 25 had accompanying survey data. Of these, 12 (48%) reported symptoms or illness of any type, and 13 (52%) did not report any symptoms. Because the study protocol did not include diagnostic testing, we were unable to confirm the presence of asymptomatic cases.

**Figure 7:**
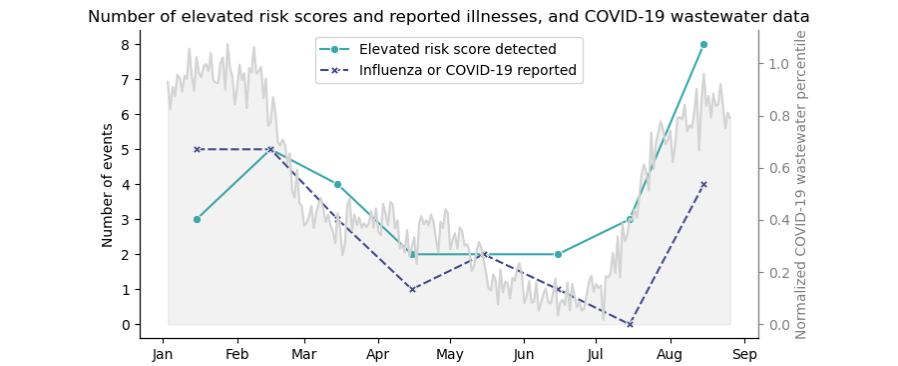
The number of influenza and COVID-19 illness and elevated risk score events observed by month and normalized US county metrics showing the percent of wastewater samples with detectable COVID-19 virus (noise added). Elevated risk scores were detected from analysis of heart rate variability-derived and activity metrics of a convenience sample of 59 participants who provided smartwatch and health survey data between January 2023 and the end of August 2023. Influenza and COVID-19 illnesses were self-reported by participants through daily surveys and may or may not have been ascertained by diagnostic tests.

We also explored the occurrence of COVID-19 and influenza cases and whether an accompanying change in risk score was observed. Based on health survey responses, 21 COVID-19 and influenza events were reported. A total of 9 (43%) participants who reported COVID-19 or influenza recorded watch data at the time of the reported illness. Of these, 6 (67%) participants had corresponding increases in risk score (above or below threshold). Table 3 summarizes the number of above-threshold risk scores detected, and influenza and COVID-19 illnesses. Figure 7 illustrates the events reported per month along with metrics of the COVID-19 virus detected in county wastewater [32].

**Figure 8:**
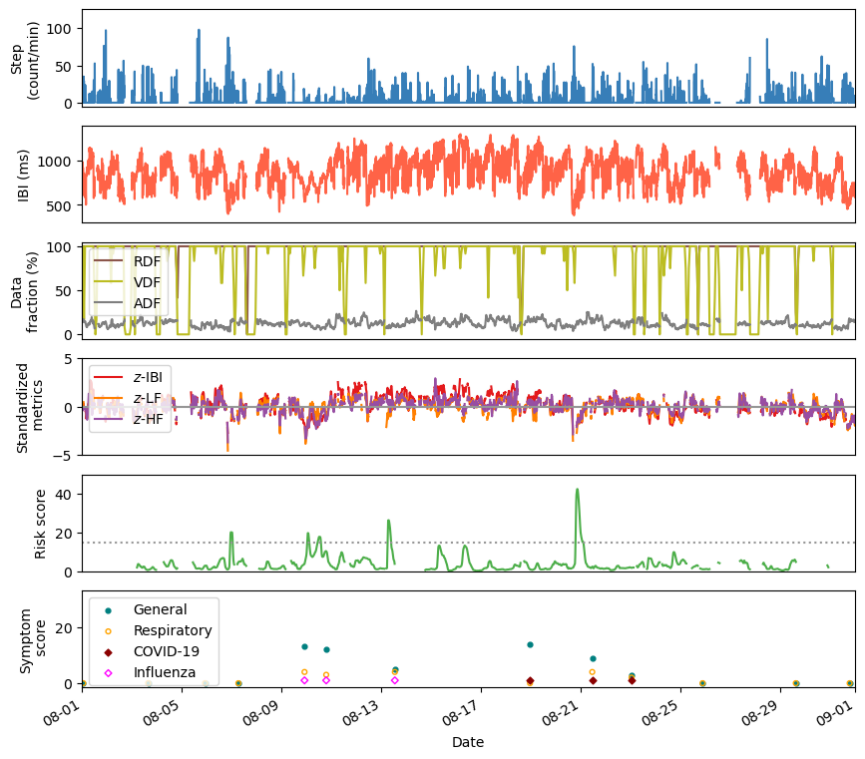
Metrics from a participant who initially reported influenza and ultimately tested positive for COVID-19 in August 2023. Elevated risk scores relative to a threshold of 15 (dotted gray line) are observed prior to and coinciding with reported symptoms. The risk scores were detected from analysis of heart rate variability-derived and activity metrics. Plots of step count, IBI, RDF, VDF, IBI ADF, standardized IBI metrics, risk score, and health survey symptom score are shown for a period of 1 month. Markers indicating self-reported influenza and COVID-19 illnesses are included alongside numeric scores of general (any type) and respiratory-related symptoms. The symptom scores were calculated from symptom ratings on a scale of 0 (no symptoms) to 3 (severe symptoms) for the following categories: allergies, runny nose, sore throat, cough, shortness of breath, fever, fatigue, headache, body ache, loss of taste and smell, and gastrointestinal symptoms. ADF: artifact data fraction; HF: log of high-frequency power; IBI: interbeat interval; IBI: interbeat interval; LF: log of low-frequency power; RDF: raw data fraction; VDF: valid data fraction; z: standardized metric.

Figure 8 shows time series plots of step, IBI, standardized HRV-derived metrics, S+ health risk scores, and symptom scores for 1 participant who reported a positive test for COVID-19 after initially reporting illness with influenza. Symptoms were reported over a period of 15 days and are shown for respiratory-specific and general (any type) symptoms. Additionally, illness reports for influenza and COVID-19 are annotated on the bottom panel of Figure 8. An elevated risk score preceding and coinciding with reported symptoms of illness is observed.

**Table 3:** Summary of self-reported influenza and COVID-19 illness or symptoms and elevated risk score events from a convenience sample of 59 individuals who provided smartwatch and health survey data between January 2023 and the end of August 2023. Elevated risk scores were detected from analysis of heart rate variability–derived and activity metrics.

Source		Count, n (%)
**Elevated risk score (n=29)**		
	Symptoms reported	12 (41)
	No symptoms reported	13 (45)
	No survey submitted	4 (14)
**Influenza or COVID-19 illness (n=21)**		
	Increase in risk score	6 (29)
	No increase in risk score	3 (14)
	No smartwatch data	12 (57)

## Discussion

### Principal Results

This exploratory study included continuous monitoring of individuals to determine HRV metrics over a baseline (healthy) window, which provides insight into long-term changes in HRV and a reference range for each metric. For the data collection and analysis, we used a standalone, vendor-agnostic modular system that is scalable to large cohorts [20].

We used the multivariate anomaly score (risk score) calculated relative to the baseline period as an indicator of exposure to stressors. The risk score was originally developed and tested in an influenza-challenge study in which participants were monitored before, during, and after inoculation, and the infection status was confirmed by a polymerase chain reaction test [16]. In this study, our objective was to assess if the risk score calculated using metrics extracted from PPG sensors during free-living conditions shows promise as an indicator of respiratory infection.

### Data Missingness and Artifacts

The observed RDF and SDF illustrate the wide variation in device compliance and study engagement in free-living conditions over long monitoring periods. The levels of device compliance and survey compliance were not necessarily related within participants (Figures 2 and 5). Technical issues with the smartwatch or use of the mobile phone app were encountered and may have contributed to reduced user compliance. These were mitigated with replacement watches, personalized technical support, and app updates. In general, using multiple data sources such as smartwatches and surveys comes with the risk of unbalanced data compliance, which should be considered in study protocols that rely on both data sources.

Along with RDF, we also report VDF and ADF. Inherent in data collection is the possibility that the VDF may be relatively low even when the RDF over a period is relatively high. This could occur in a situation where the watch is recording data but is not actually being worn. For example, in some of these cases, an increase in IBI ADF can indicate artifactual data recording (Figures 2-4).

### Cohort Observations

The interpretation of risk algorithm performance depends fundamentally on wear compliance and quality of ground truth information, in this case, symptom and illness reporting compliance. A total of 12 (57%) influenza and COVID-19 cases reported did not have accompanying smartwatch data. Of the 9 (43%) that did have watch data, 6 (67%) were observed to have an increase in risk score. Influenza and COVID-19 infections were identified from self-reports rather than by diagnostic tests; and thus, there may have been cases that were not reported and reported cases that were not in actuality influenza or COVID-19.

We explored the prospective use of health anomaly detection based on a previously determined parameter threshold for this algorithm [16]. A total of 12 (48%) elevated risk score events relative to this threshold that included survey data also reported symptoms or illness. The choice of value for the threshold parameter was based on the results of a previous study that used ECG measurements of patients with confirmed infection status, and thus may not be optimally tuned for this PPG sensor data set.

The small sample size and lack of diagnostic tests to confirm self-reported illnesses limited statistical analysis and the ability to explore threshold optimization in this study. Similarly, we did not have enough cases to investigate the application of the algorithm to health anomalies other than influenza, the characteristics of which may or may not be adequately detected. We did observe similar trends in the number of elevated risk scores and number of reported influenza and COVID-19 cases per month, which were qualitatively similar to trends in COVID-19 virus detected in county wastewater (Figure 7).

Approximately 47% of influenza and 40%-45% of COVID-19 cases are asymptomatic [33,34]. Without a rigid testing protocol in our study design, and without symptoms to prompt participants to seek testing, we were unable to confirm asymptomatic cases. Figure 8 illustrates a symptomatic infection case that was initially presumed to be influenza but was later confirmed to be COVID-19 through testing. This example highlights the importance of ground truth data for research interpretation and that, in the absence of health testing, illness presence or type is not necessarily known.

Vaccination status for influenza and COVID-19, including COVID-19 boosters, was self-reported at the time of study onboarding, which preceded the start of the monitoring period reported in this paper by 2 months. Updates to vaccination status were not requested during the study. Therefore, it is possible that additional vaccinations were obtained by participants and not recorded in the cohort characteristics listed in Table 1.

### Incorporation of Other Algorithms and Sensors

The data collection and analysis platform can incorporate other algorithms that use HRV metrics obtained from physiological sensor data to detect illness. Similarly, other physiological sensors could be chosen for data collection. The modular nature of the software platform and the use of transparent, open-source routines for data cleaning and metrics extraction, bypassing algorithms proprietary to device vendors, supports the incorporation of other analytical approaches or wearable devices.

### Limitations

We have discussed the limitations of cohort size and ground truth uncertainty for the assessment of illness detection in this study. Additionally, some technical challenges were reported by participants, which were mitigated by watch replacements and smartphone app updates. A further shortcoming is the lack of racial diversity in the study cohort. Recent research indicates that the accuracy of HR measured by most leading wearable optical sensors is not significantly affected by skin tone, although devices themselves vary in their measurement accuracy, particularly during activity [35]. A 2019 survey of 4272 individuals reported only modest differences in smartwatch use across race, ethnicity, and sex, but larger disparities based on income level [36]. This has raised the additional concern of socioeconomic bias in wearables health monitoring [37]. Algorithm biases based on sex and age have also been reported [19]. To improve health equity, future studies should strive to include a more balanced representation of participants.

### Comparison With Prior Work

Previous research reporting on the use of wearables sensors for long-term health monitoring has obtained data processed by vendor-provided analytics, included a cohort based heavily on a smartwatch or smart ring ownership or on active military status, or performed analysis retrospectively [15,17-19]. To our knowledge, this is the first study to collect high-resolution IBI data recorded from the device in a community-based civilian sample in free-living conditions for prospective analysis that does not require accessing the device vendor cloud and is compatible with near real-time monitoring.

Other long-term monitoring studies have incorporated surveys along with wearables data [15,17-19], but to our knowledge, studies have not reported metrics for detailed quantification of data collection, missingness, and quality for the wearable and survey data. Neglecting to wear the device during periods of illness, which we observed in our cohort, has also been reported in a previous study in which only 32 of 114 (28%) participants wore their devices around the time of infection [18]. A study of 30,529 participants spanning 11.5 weeks in the spring of 2020 reported that only 8.7% of participants who experienced symptoms followed up with a COVID-19 test, reinforcing the reality that diagnostic testing may not always be available or used when monitoring population health [37].

### Conclusions

We have described the application of a data collection and analysis pipeline for continuous wearable–based physiological monitoring and health risk score assessment under free-living conditions over a period of 8 months. This study used commercial-off-the-shelf sensors and open-source algorithms that provided updated health scores every 5 minutes. High-resolution data were collected directly from the smartwatch hardware and processed automatically through an integrated software platform. The platform architecture is modular, and different sensors or algorithms could be incorporated. Data quality was quantified for smartwatch data using metrics for raw, valid, and artifact data fractions and for health survey data. Study engagement was generally sufficient to calculate health risk scores, although inconsistent survey inputs limited confirmation of illness in relation to elevated risk scores or of physiological changes in relation to reported illness in many cases. The risk score calculation shows promise as an indicator of respiratory infections but needs to be validated in a large-scale study, in which the illness is confirmed by laboratory tests. To our knowledge, this study represents the first to collect high-resolution, real-time IBI and step count data directly from the device (bypassing the device-vendor cloud and vendor-provided analytics) during long-term monitoring of a community population in free-living conditions and analysis of the data to assess acute respiratory health anomalies in real time.

## Abbreviations

ADF: artifact data fraction
ECG: electrocardiogram
HF: log of high-frequency power
HR: heart rate
HRV: heart rate variability
IBI: interbeat interval
LF: log of low-frequency power
PPG: photoplethysmography
RDF: raw data fraction
REDCap: Research Electronic Data Capture
SDF: survey data fraction
VDF: valid data fraction

## Data Availability

The data analyzed in this study are not publicly available due to restrictions of the Defense Advanced Research Projects Agency (contract 140D63-19-C-0023) but are available from the corresponding author on reasonable request.
